# Modeling of Microstructure Evolution during Deformation Processes by Cellular Automata—Boundary Conditions and Space Reorganization Aspects

**DOI:** 10.3390/ma14061377

**Published:** 2021-03-12

**Authors:** Łukasz Łach

**Affiliations:** Faculty of Metals Engineering and Industrial Computer Science, AGH University of Science and Technology, Mickiewicza Av. 30, 30-059 Krakow, Poland; lach@agh.edu.pl

**Keywords:** cellular automata, geometry of the cellular automata space, boundary conditions, space reorganization, materials science

## Abstract

Cellular automata (CA) are efficient and effective numerical tools for modeling various phenomena and processes, e.g., microstructure evolution in plastic working processes. In many cases, the analysis of phenomena can be carried out only in a limited space and on representative volume. This limitation determines the geometry of CA space hence boundary conditions are very important issues in modeling. The paper discusses different boundary conditions that can be applied to modeling. Taking into account the transformation of the modeling space, the model should allow the selection of boundary conditions. The modeling of certain phenomena and processes is directly related to changes in the geometry of a representative volume and therefore may require changes or reorganization of the modeled CA space. Four reorganization options are presented: halving, cutting and bonding, doubling, and straightening. A choice of boundary conditions may depend on particular space reorganization as used for the modeling of microstructure evolution. A set of decision rules for selecting space reorganization options taking into account the changes of CA shape and sizes is also presented. The modeling of flat and shape rolling processes utilizing some of the described techniques is shown.

## 1. Introduction

The numerical modeling of various phenomena and processes is based on different schemes, tools, and methods. One of the more frequently applied methods is cellular automata (CA). Dynamic growth of interest in this method in modeling of many phenomena and processes is observed. Complex, CA-based models are used in many areas of science.

Materials science and metallurgy are important areas of cellular automata application. CA allow the creation of digital material representations [1]. Furthermore, the cellular automata technique was used for modeling solidification processes [2] and dendritic growth in the mesoscale [3], simulating the decomposition of austenite into ferrite [4], transformation of austenite into ferrite and perlite [5], dynamic recrystallization [6], static recrystallization with recovery [7]. CA are also widely used for modeling severe plastic deformation [8], rolling processes [9,10], grind-hardening processes [11], and additive layer manufacturing [12,13]. Frontal cellular automata (FCA) is a relatively new modeling tool that creates new possibilities of numerical analysis in comparison to the classical form of cellular automata algorithm. Frontal cellular automata allow modeling three-dimensional processes within a reasonable time. Three-dimensional FCA computational time is similar to the computation time of 2D CA based on the classical algorithm. Advantages and principles of this kind of automata can be found elsewhere [14].

The cellular automata can be considered as a system defined by various parameters, for example, cell state, neighborhood, and transition rules. Space geometry, which is a very important element characterizing the CA, is directly related to boundary conditions. An analysis is often applied to the very small representative volume of investigated material and only a selected part of the whole specimen is modeled. Boundary conditions are directly connected with the selection of the cellular automata space geometry. They should eliminate the edge impact because all the elements (cells) in the CA space should be in the same, equal conditions. The cell located on the edge of the space should have the same conditions and neighbors as other cells located in the middle of the space; as it is surrounded by the same material, the phenomena and processes occurring within the cell are the same as in any other place. Appropriate boundary conditions have a significant impact on modeling results. Another important modeling issue, directly connected with boundary conditions, is space geometry. Consideration of real deformation of the modeled cellular automata space may lead to significant distortion of the whole space and cells. Furthermore, the deformation has an impact on the level of anisotropy of the CA modeling space, i.e., the cell size ratio may be too large, calculations slower and the quality of the obtained structure unsatisfactory. Taking these conditions into account, the structure of cellular automata should be reorganized. Such actions have a direct impact on boundary conditions, which may require changes. Another important element related to space geometry is an appropriate choice of the cell grid. Lattice geometry is one of the most important parameters defined for the CA. A grid is an array of cells each of which can contain a value (or “be in a state”) from a set of possible cell states. Depending on the task, grids can be one-dimensional, two-dimensional, or multidimensional. For 1D automata, the lattice is a linear array of cells. For 2D, the grids composed of regular polygons, i.e., triangular, square, hexagonal are often used. The grid can also be regular or irregular, finite or infinite, unchangeable or changeable in shape and size. Square geometry is usually applied because it is easy to define its neighborhood. However, if a more isotropic interaction is required, the use of hexagonal geometry may be a better choice. Three-dimensional offers more possibilities. As before, cubic geometry is the most frequently used. The majority of results obtained with the use of CA were based on regular lattices of cells. Complicated geometry and/or stress concentration regions may require the application of highly irregular grids. Many studies linked with the application of regular grids can be mentioned such as a 3D model for the prediction of dendritic grain structures formed during solidification using a regular lattice of cubic cells [15], recrystallization microstructure prediction of a Hot-Rolled AZ31 Magnesium Alloy Sheet in combinations with 2D square lattice and Neumann’s neighboring rule [16], computing two-dimensional elastodynamic response on arbitrary domains by triangular cellular automata [17], or modeling the growth of dendritic electroless silver colonies using hexagonal cellular automata [18]. In the orthogonal coordinate system, due to its symmetry, the rectangular grid is generally preferred. To avoid ambiguity linked with the use of rectangular grids, hexagonal grids with a simpler and more symmetrical nearest neighborhood can be used. Random [19] or irregular [20] grids are also used for modeling. Application requirements and objectives should determine the selection of an appropriate grid.

The paper presents selected feasible space boundary conditions, where the changes in geometry are not necessary, or where changes have not a significant impact on the space topology, and basic rules for boundary application conditions where space reorganization becomes necessary. Different ways of space reorganization have been developed and shown. The choice of the reorganization option depends on requirements and assumptions regarding space sizes and representative modeled volume. The options include cutting, cutting and bonding, doubling. When high distortion is applied to space, a different option for cellular space straightening is used. A set of decision rules with respect to the choice of the reorganization option is presented in the following sections. Finally, the application of the presented techniques for modeling of flat and shape rolling processes was also shown.

## 2. Boundary Conditions

The literature on cellular automata modeling distinguishes two basic types of boundary conditions, i.e., open (referred to as semi-open or open depending on properties) and closed [21,22]. These two types of boundary conditions also allow their different combinations. It is important for the modeling to determine which conditions should be applied to a given issue. The open and closed boundary conditions are usually used for CA modeling of microstructure evolution in different processes. A review of the literature in the field of CA applications to the modeling of microstructural phenomena shows a visible advantage of periodic boundary conditions. However, their application is not strongly justified. In general, the use of periodic conditions in the simulation of the microstructure evolution is explained by the creation of an illusion of infinite space with a repeating pattern. In addition, there are two basic differences between open and periodic conditions connected with boundary impacts. Grain size in the microstructure obtained under the periodic boundary conditions is independent of grain location which influences the grain size in microstructure obtained under the open boundary conditions; the fine grains near the boundaries become smaller and the coarse grains become greater. Another very important difference is the influence of open conditions on the recrystallization kinetics. Lack of “edge impact” due to the equivalence of all cells constitutes the basis for more realistic simulations of the processes under periodic conditions. This is the main reason why the cellular space is closed and periodic conditions are used for example for the modeling of the grain growth during the crystallization, or recrystallization, or simulation of other physical phenomena. Connecting the opposite sides of the space eliminates the influence of the edges on the growth process. In this section, four basic boundary conditions were considered: semi-open, open, periodic (closed), and combined. The use of open and closed conditions is widely described in the literature. The presented semi-open conditions, periodic conditions with displacement and combined conditions applied to the modeling of plastic processes constitute newly proposed solutions, which can be used in the modeling involving CA space reorganization. With regards to unchanging topology, only semi-open conditions do not always meet the conditions imposed by the simulation (for example, concerning the kinetics of the processes). With respect to varying topology, the most useful conditions are periodic conditions with one direction displacement equal to half of the space length. More precisely, these conditions can be also defined as combined conditions with the properties of periodic conditions with displacement. Such conditions allow basically any reorganization. Combined conditions also admit different conditions of neighboring grains and changes in conditions during the simulation process.

### 2.1. Closed and Open Boundary Conditions

**Figure 1:**
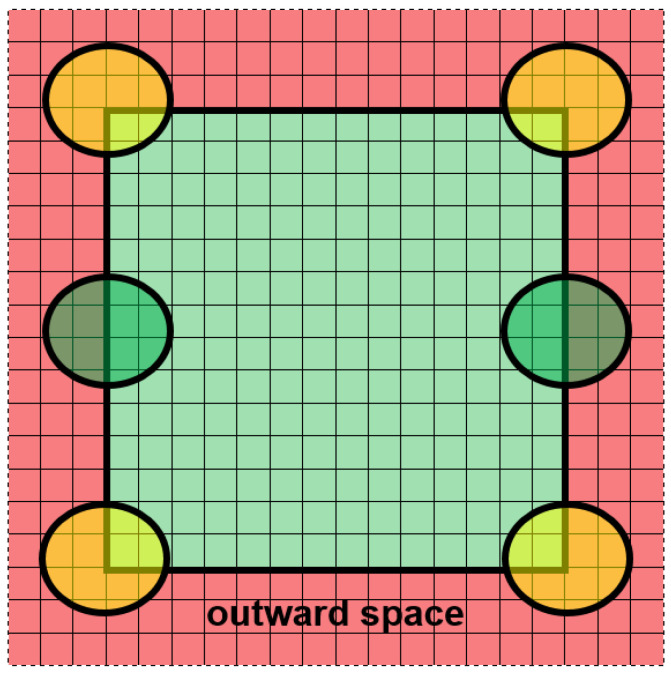
Periodic boundary conditions.

**Figure 2:**
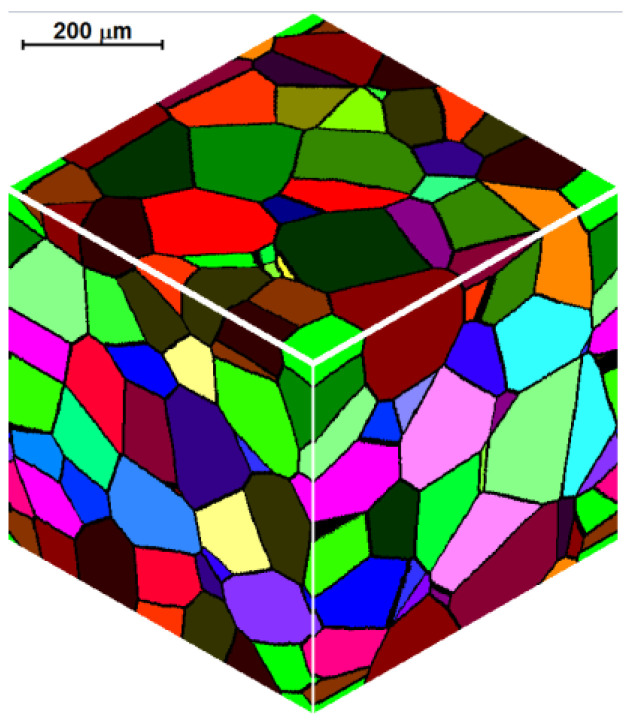
Example of microstructure with periodic boundary conditions.

The boundary condition, also referred to as periodic condition, is the first condition type. It is used, among other things, for the simulation of the recrystallization process of hot deformed austenite of TRIP steel [23], in the modeling of grain coarsening and refinement during the dynamic recrystallization of pure copper [24], in two-dimensional CA with the quadrilateral element, or in the modeling of dynamic recrystallization microstructure evolution for 316LN stainless steel [25]. This type of boundary connects the boundaries of the cellular space and allows introduction of the dependence between their different parts. The opposite boundaries can be “glued” together. All space cells with periodic boundary conditions are in the same equal neighborhood and no external influence (from outside the space) can be observed because there are no space boundaries. Grains, which grow up to nominal boundaries of the space, continue to grow and appear on the opposite side, as shown in Figure 1. Figure 2 represents the application of periodic boundary conditions to create an initial microstructure with grains growing in the shape of the sphere. CA space contains *n_x_* × *n_y_* × *n_z_* = 300 × 300 × 300 cells with the representative volume of *a_x_* × *a_y_* × *a_z_* = 500 × 500 × 500 μm^3^ and includes 100 grains.

The most complicated conditions are full-open boundary conditions as they involve the disappearance of any dependence between the opposite sides and the elimination of the edge impact on the space edge. The full-open boundary conditions were used for example in CA analysis of primary static recrystallization in low carbon steel [26]. Under the full-open boundary conditions, the impact of external areas (outside the modeling space) is balanced by the influence of cellular automata on these external areas. This interaction is symmetric. The full-open condition can be described as follows: when the boundary is achieved by the growing grain, another grain will appear not exactly on the opposite side only but also on the arbitrary location. It is no longer the same grain. The sides where the new grain grows into the cellular space and appearance coordinates (including the distance from the surface) are chosen randomly.

**Figure 3:**
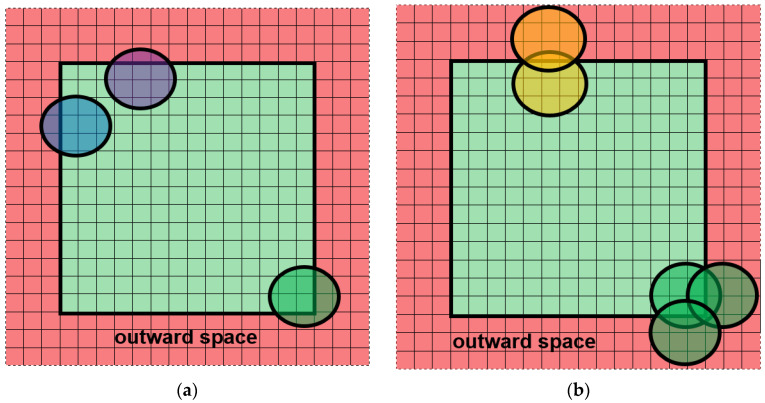
Semi-open boundary conditions: (**a**) growing grains go outside the space, (**b**) grains inside the modeling space.

Semi-open boundary conditions are a specific variant of open conditions and a new proposition with respect to the modeling of plastic processes. The imbalance is the main weakness of the semi-open boundary conditions: boundaries are opaque in one direction (inward) and transparent in the other (outward). The external grain cannot grow inward; however, the internal grains can grow outward. Contrary to the closed ones, the semi-open boundary conditions are characterized by fully independent opposite sides of the space. It means that nothing exists outside the space (behind the defined boundaries). For example, when the grains (the blue, green, and purple grains in Figure 3a) reach the boundaries of the modeled space (light green), they grow further outward (light red) without CA receiving any information about it. The sizes of such grains are unknown and information about their shape is incomplete. A situation like this can also be considered in a slightly different, more artificial way as in Figure 3b. The grains (yellow, green) are considered to be fully inside the modeling space but there are the same grains outside the space located symmetrically in relation to the boundary.

**Figure 4:**
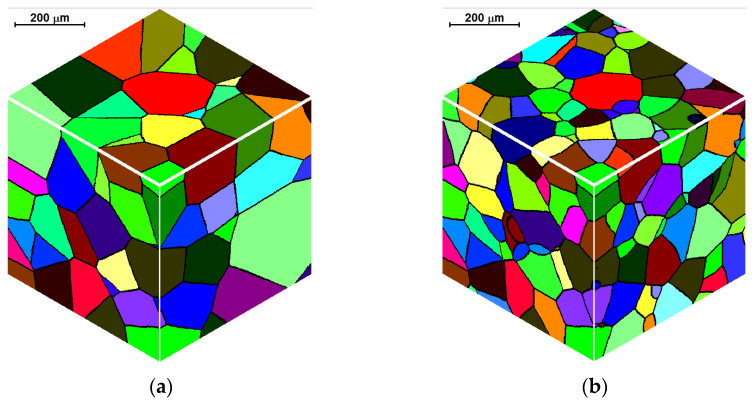
Initial microstructure with semi-open (**a**) and full-open (**b**) boundary conditions.

Examples of three-dimensional microstructures obtained under semi-open and full-open boundary conditions are presented in Figure 4. Modeling conditions are the same as in the previous example. It can be seen that the microstructure, created under the semi-open conditions, has greater grains near the space boundaries, which is consistent with the characteristics of these conditions described above.

### 2.2. Periodic Boundary Conditions with Displacement

**Figure 5:**
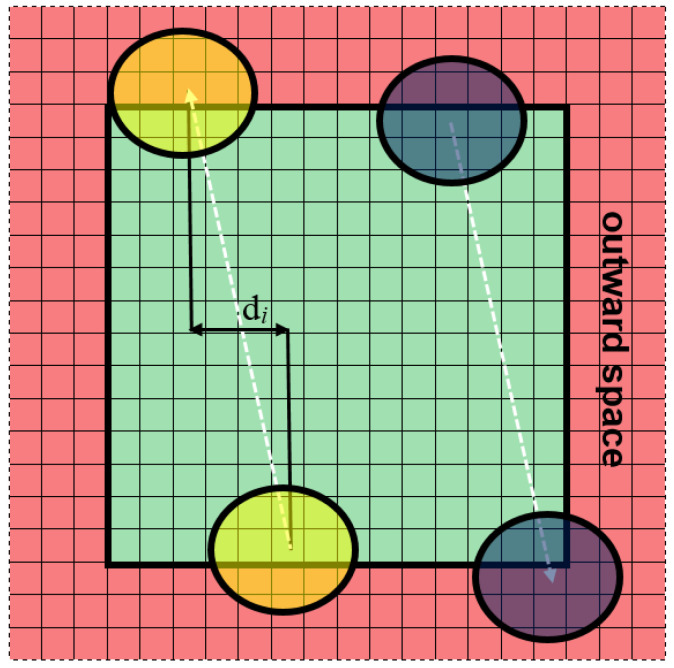
Periodic boundary conditions with displacement.

An analysis of multistage processes with considerable geometrical distortions is directly associated with the necessity of space reorganization. More complex periodic boundary conditions with displacement (sometimes called helicoidal) can offer some advantages to this kind of problem. The helicoidal boundary conditions are described in the literature, however, in relation to other issues [27]. The use of periodic boundary conditions with displacement for the modeling of microstructure evolution during plastic processes involving space reorganization represents a new approach in this field. The space can be closed with the displacement of the bonding planes or lines. The scheme explaining such conditions is shown in Figure 5. A neighboring cell is placed on the opposite side of the space not straight but with some displacement. The vertical crossing of the boundary means a change not only of vertical but also horizontal coordinates. The displacement can be set as constant for the entire boundary.

**Figure 6:**
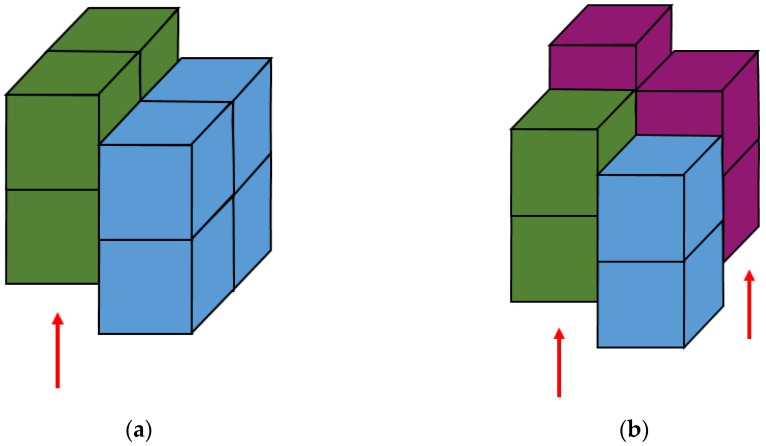
Boundary conditions with displacements in three-dimensional space: (**a**) one displacement, (**b**) two displacements along the one axis.

**Figure 7:**
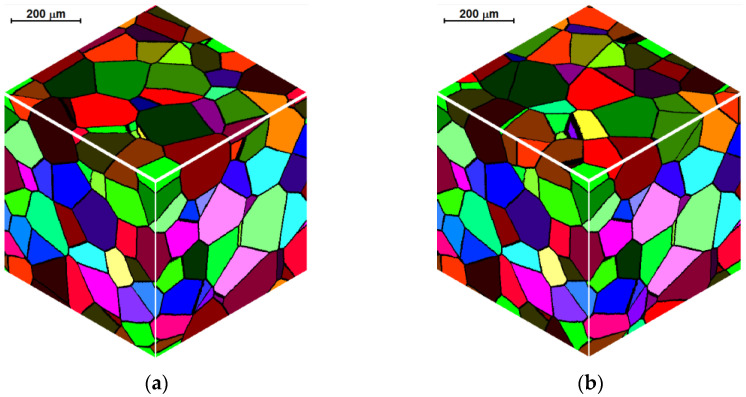
Three-dimensional microstructures with periodic boundary conditions with displacements corresponding to the displacement schemes presented in Figure 6: (**a**) one displacement, (**b**) two displacements along the one axis.

This application is relatively little-known and rarely used in practice. In 2D space, only two displacement variants can be realized, i.e., towards the *x* or *y* axes. The simultaneous use of both displacements in the 2D space is inadmissible. The overall number of displacements, which can be applied to 3D space is six. There are two main orthogonal directions of displacement in each of the three opposite planes of the space. Figure 6 represents two variants of boundary conditions with one or two displacements in 3D space (each cuboid represents the same CA). There are six groups available in the 3D space. Each group contains one to six different variants of axis and plane permutations. Examples of microstructures obtained in the space under the periodic boundary conditions with displacement in different directions are shown in Figure 7. The initial conditions for the formation of the microstructures are the same as for the previously presented variants.

### 2.3. Combined Boundary Conditions

**Figure 8:**
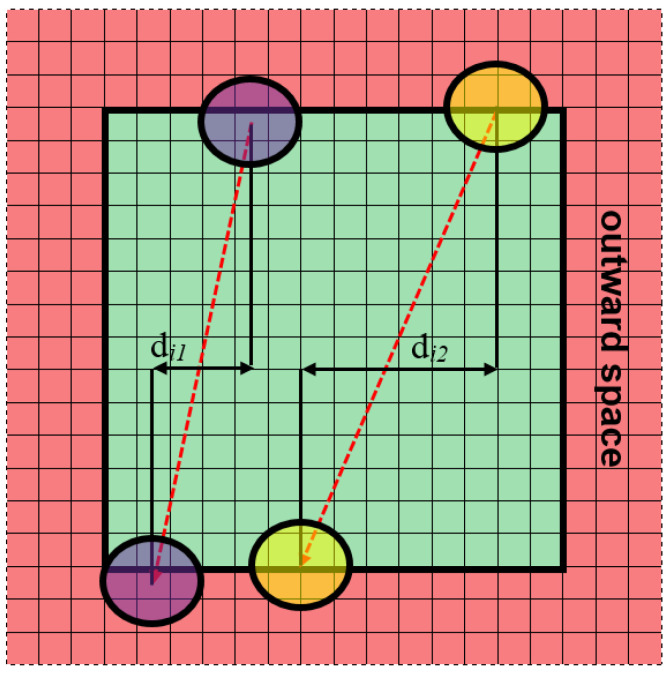
Combined boundary conditions.

The same impact as for the periodic boundary conditions with displacement can be obtained where equal displacements are assigned not to the space but to every grain in the space without displacement in which case the displacements are interpreted differently in either approach. It is no longer the property of the space, but of the grains. Furthermore, different displacements can be assigned to different grains. Figure 8 shows a variant with different displacements for two grains. After crossing the space boundary, the grains can appear in arbitrary places representing combined, instead of periodic, conditions. This solution can also be treated as new in the context of the application to the modeling of microstructure evolution with space reorganization.

### 2.4. Application of Boundary Conditions

Considering a one stage process and single simulation, whether periodic boundary conditions with or without displacement or fully open conditions will be used for the calculations is irrelevant. Taking into account the computational effort and time required to develop proper software, it is rational and more advantageous to use the simplest algorithms possible allowing adequate modeling. In this case, any simpler variants are unquestionably associated with the use of periodic conditions without displacement. In more complex calculations related, for example, to the analysis of multi-stage processes, advantages derived from more complex boundary conditions (e.g., the periodic conditions with displacement) become evident. The choice of a given type of boundary conditions has a direct impact on the modeling results obtained with the use of space reorganization methods which are presented in the following section. With regard to the halving process (reducing the dimensions of the model space), the boundary conditions can be defined freely because their type does not significantly influence the results. When increasing the space dimensions by, for example, doubling one or several dimensions, the boundary conditions influence the repeated step of the microstructure pattern. In the conditions without displacement, this step will be the shortest, whereas, in the conditions involving displacement, the step will double in one or two directions. The change of the shape of space causes the boundary conditions to significantly influence the microstructure after the reorganization. The periodic boundary conditions with displacement have a noticeable advantage over other conditions if one dimension is shortened and another dimension increased, in which case the periodic conditions without displacement space would be first divided with one part rejected, while the other should be doubled and bonded. As a result, a repeating pattern will be forced in one direction while semi-open boundary conditions in the other. This situation can be easily avoided by using periodic conditions with displacement. After dividing the space, both parts can be bonded. Analyzing the cellular automata space straightening algorithm, the final boundary conditions will be almost certainly half-open. However, there will still occur a difference in the length of the repeating step of the same microstructure pattern. The periodic conditions with displacement will also have some advantages in this case. Taking into account the modeling of a multi-stage flat rolling process with huge overall deformation, the periodic boundary conditions with one displacement appear to be most rational to apply (Figure 6a). It is a good solution to apply periodic boundary conditions with two displacements in the modeling of microstructure evolution during the shape rolling process (Figure 6b).

## 3. Space Reorganization Methods

The study of substantial deformation in one or multiple-stage deformation processes often shows changes in the whole space of cellular automata and in each individual cell. For example, the elongated flat space may not be suitable for modeling due to its small thickness. It is more difficult to keep space isotropy. Calculations based on cellular automata may begin with the cuboid cells (but not necessarily). They should not preserve the same shape and sizes during the simulation of the process with deformation. The deformation has an impact on not only the cell geometry but also the level of anisotropy of the modeled space and the representative volume. A large deformation leads to the distortion of the space and cells and requires space reorganization. The reorganization in cellular automata is similar to the “remeshing” widely used in FEM codes [28], and improves the accuracy of the calculation when geometrical parameters of the cells become unacceptable.

The CA reorganization with the constant number of cells in modeling space can be carried out in three different ways; the choice depends on the requirements and assumptions underlying space sizes and representative volume. The first one may be used when cellular space can be reduced (halving); the second one, when the modeled volume is not changed (cutting and bonding); the third one, when the cellular space should be increased (doubling). There are some cases where none of these methods can be applied and a more universal space straightening method has to be used. The methods of space reorganization discussed in the following sections represent new solutions in the modeling of microstructure evolution during plastic deformation processes considering real deformation for the cellular automata space.

The reorganization methods include operations on space and cells. On some occasions, the sequence of these operations can be arbitrary while on others, it is fully prescriptive. The developed reorganization methods can be useful when deformation is significant and uniform. In the case of the regular grid and uniform deformation, calculations are common for all the cells and the whole space. Each element has the same shape and it suffices to calculate changes of one cell to determine the coordinates of the centers or vertices of all cells. Using the strain tensor in the calculations, new sizes and shapes of the cell can be obtained. These deformation conditions can be taken into account not only during the modeling of dynamic recrystallization or flat rolling, where directions of the main strains are consistent with the axes of the cellular automata space, but also in the arbitrary process with uniform deformation.

### 3.1. Halving of the Modeling Space

**Figure 9:**
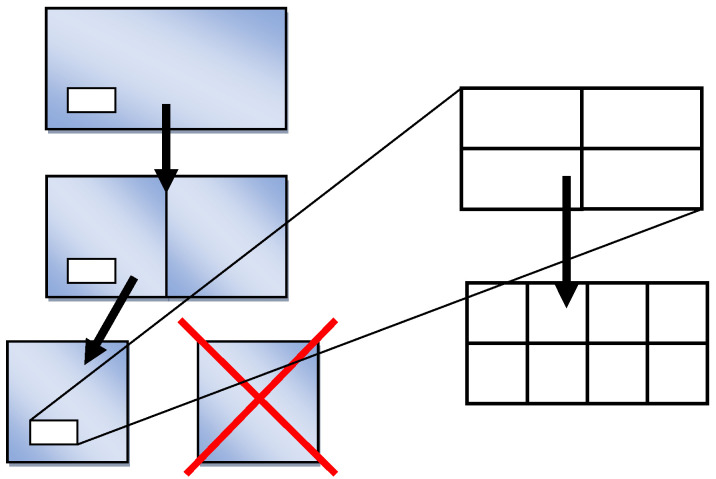
Scheme of cellular space halving.

The halving method relies on the cutting of representative space and consists, primarily, of the cell–cell division operation. The cell side size (or the representative volume) ratios are checked after the deformation, or before the next deformation. All space is halved if the ratio nearly reaches 2.0. For further simulations, the first half of the cellular space is left whereas the second half is removed from the model. In the remaining part, each cell is also halved (Figure 9). Both halves of the cell inherit all properties of the parent cell and become new cells. The colored items in Figure 9 represent the whole modeling space while the white items several cells.

Removal of one half reduces one size of the space thus decreasing its volume and number of cells. After the halving, the shape of the cell and space should be closer to square (or cubic); the remaining cells are divided into two and the number of cells after the halving is the same.

This method can be used, for example, when deformation is coupled with the microstructure refinement or during the modeling of the rolling process. Space halving is realized after the deformation and recrystallization when the number of grains from cut to cut has not decreased significantly.

The halving under half-open, combined, or full-open boundary conditions does not require an additional operation. However, the periodic boundary conditions lead to the malfunction of the boundary conditions after the cutting. This problem can be solved by the use of combined boundary conditions, which should be applied to the grains, not to space. Then, semi-open boundary conditions are applied to the grains which were cut by way of the halving, while periodic conditions remain the same for the uncut grains. Thus, the semi-open conditions are applied to all new boundaries of the modeled representative volume that appeared after the halving. During the further modeling, they can initially evolve into partly-open, partly-periodic to eventually change into fully periodic conditions because every internally growing grain, which moved to the boundaries of the CA space, has periodic boundary conditions.

**Figure 10:**
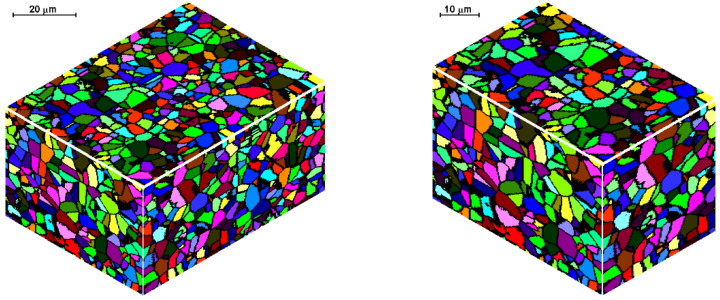
Reorganization of space by cutting in one direction.

Cutting operation can be repeated for any directions, or coordinates. Figure 10 shows halving in one direction.

### 3.2. Cutting and Bonding

This reorganization method contains operations on space and cells which can be fulfilled in an arbitrary sequence.

**Figure 11:**
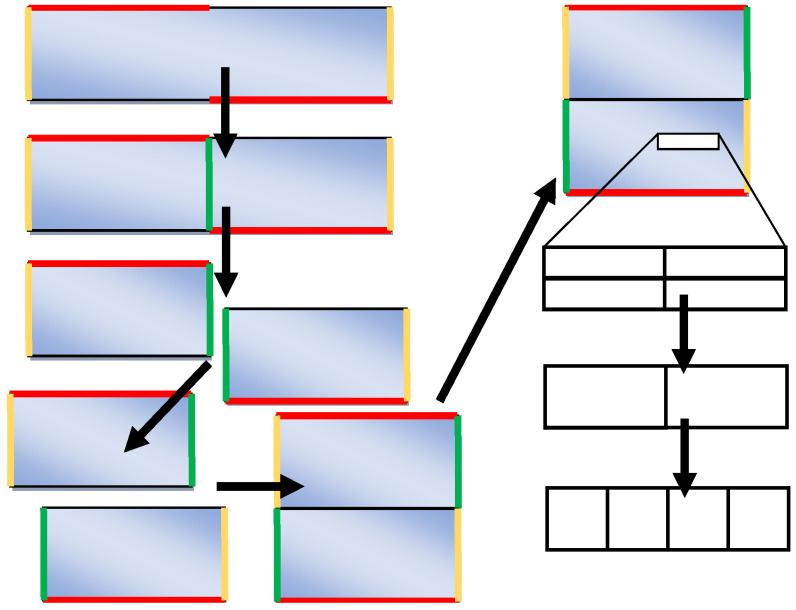
Modeling space cutting and bonding and boundary conditions before and after space reorganization.

The operation on the space includes cutting and bonding, i.e., cutting the space into two equal parts and putting them together with a changed configuration. One size of the space is halved while the other is doubled without changes in the volume of the whole space. This method can be applied where the number of grains remains the same; however, space elongation requires reorganization: the sizes of the space are changed—the largest size is reduced and the smallest one is increased (Figure 11).

The operation of the cells consists of two steps: bonding and cutting. In the first step, the paired cells should be bonded so that the shape of new cells is closer to a square or cube. Subsequently, every new cell is divided into new twos which become even closer in shape to the square or cube. Cells that are part of different grains are combined taking into account the neighboring cells. Thus, to be subsequently increased, the number of cells is first decreased twice. As a result, the number of cells remains the same. The sequence of these steps (i.e., cell bonding and then division) is important because the division of cells (i.e., multiplication of the number of the cells) requires more computer memory and the other sequence can lead to exceeding the available memory. The operation on the cells increases the number of cells in one direction and decreases it in another. However, the number of the cells in both modified directions remains the same as before reorganization if both operations on the space and cells are carried out.

With regard to this reorganization and space direction (which should be doubled), periodic boundary conditions with displacement should be applied before modeling. The periodic boundary conditions without displacement should be attached to other boundaries. After the reorganization, the boundaries with displacement become the boundaries without displacement and vice versa, as can be seen in Figure 11. The displacement in this scheme is equal to half of the space length.

**Figure 12:**
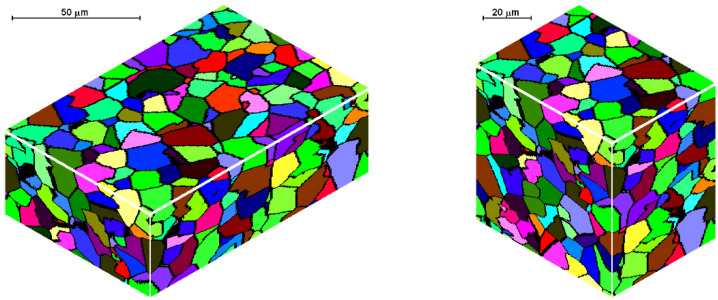
Reorganization of space by cutting and bonding.

An example of 3D space cutting and bonding is presented in Figure 12. After the operation, the parts perfectly fit into one another and it is difficult to find the connection line in the newly reorganized space.

### 3.3. Space Doubling

**Figure 13:**
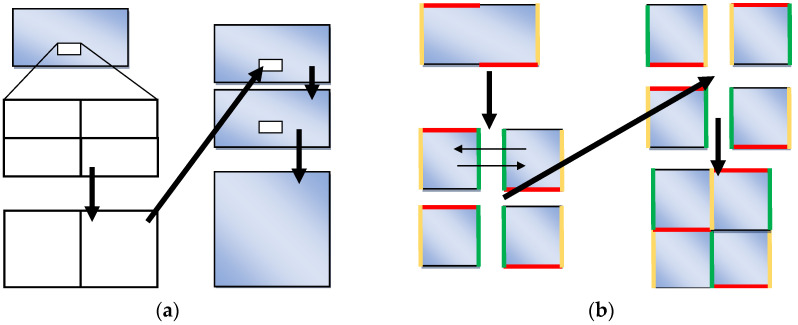
Doubling of space with periodic boundary conditions: (**a**) without displacement, (**b**) with displacement.

**Figure 14:**
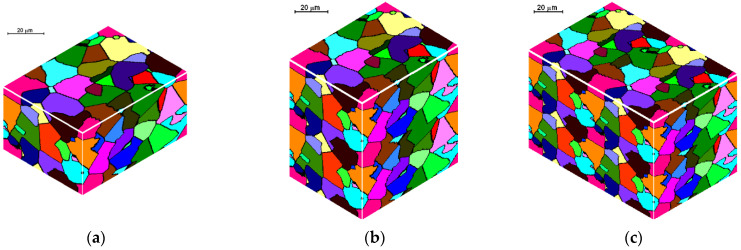
Example of space doubling: (**a**) initial structure, (**b**) structure after the doubled in the vertical direction, (**c**) structure after the doubled in the vertical and horizontal directions.

A connection between two parts of the space by way of the doubling method requires that the structure should be the same in the location of joining. The easiest way to achieve it is to use periodic conditions without displacement, as shown in Figure 13a. This method can be also applied to the space with periodic conditions with displacement (Figure 13b). The doubling can be repeated several times. Simple space doubling leads to the doubling of the number of cells; however, computational requirements can limit that number. Thus, every cell pair should be combined into one. This variant of reorganization can be realized through joining, as shown in Figure 13a, with white objects representing the cells; the number of cells is reduced twice. Then the CA space is copied into the second part, which is bonding with the first part—the original CA space. In the case of periodic boundary conditions with displacement, the cells are combined as before and the first part of the new space is filled. The second part is filled with displacement. The result of reorganization is a space with periodic boundary conditions without displacement (Figure 13b). An example of doubling is shown in Figure 14.

### 3.4. Straightening of the Cellular Automata Space

**Figure 15:**
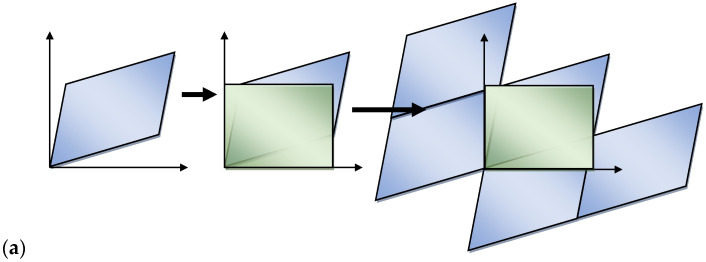

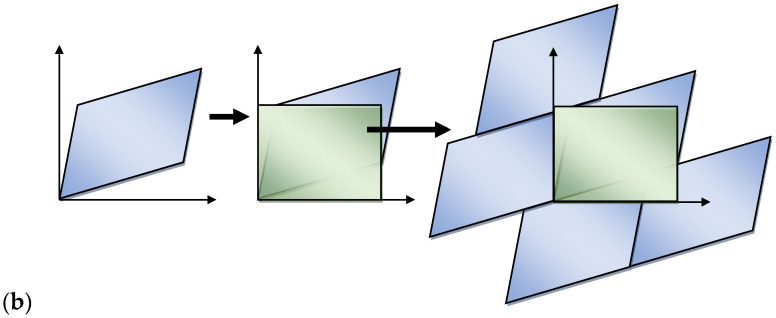
Replacement of the distorted cellular automata space with periodic boundary conditions through a rectangular space: (**a**) periodic conditions without displacement, (**b**) periodic conditions with displacement.

The reorganization variants presented in the preceding sections are related to the cases in which directions of the main strains correspond to the axes of the cellular automata space or are close to them. In the multi-stage deformation, it is not always possible to preserve the cuboid shape of the cellular automata space; it may become an oblique parallelepiped with the cell walls and the whole space inclined. If the shape distortions become too big, it is necessary to “straighten” the oblique parallelepiped to the cuboid shape. Such a reorganization can be fulfilled only for spaces with periodic boundary conditions with or without displacement (Figure 15).

**Figure 16:**
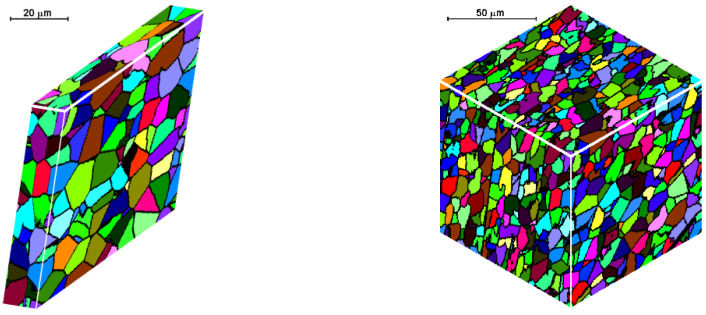
Straightening of cellular automata space.

Periodic boundary conditions allow easy unlimited replication of the cellular automata space and the creation of a new expanded space with many copies of the same repeated image. In this method, a new space can be written into the expanded space without any restriction of the shapes and sizes. Examples of such operations are shown in Figure 15. Figure 16 represents space straightening and the microstructure before and after the straightening.

### 3.5. Automatic Selection of Reorganization Method

**Figure 17:**
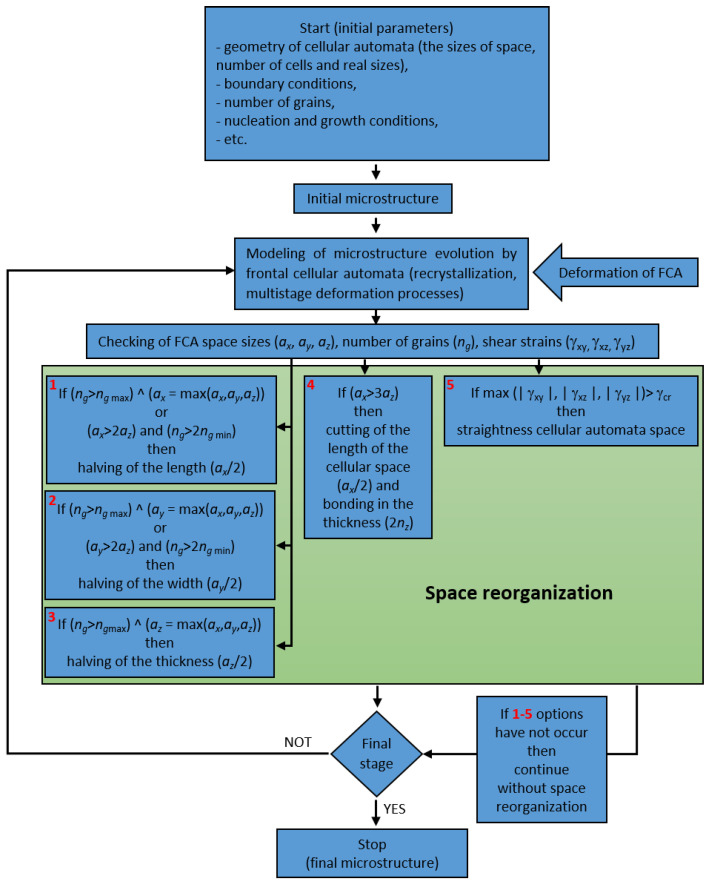
Block diagram of automatic selection of the space reorganization method.

Reorganization methods are varied and their choice depends on boundary conditions at the beginning of the modeling. It is better to choose an appropriate method before the modeling but it is not always possible. It is related, for example, to the fact that there is an optimal number of grains for the selected space sizes. It can be assumed that the maximum number of grains in 3D space is equal to the number of cells along one dimension of the space and the minimum number of changes occurring at the intervals of 25–40 grains. Significant increases or reductions in the number of grains diminish the modeling efficiency. Therefore, modeling results influence the choice of space reorganization method. Microstructure fragmentation will lead to a significant increase in the number of grains, reduction of their volume and increased adverse effects of space discretization. That is why, in this case, choosing the halving option is desirable. On the other hand, the halving option maintaining, or slightly reducing, the average grain size, will lead to the reduction of the number of grains below a rational value and perhaps up to a point where the grain will extend from one edge to the other. A solution may be to repeat the calculation with changed boundary conditions and appropriate order of space reorganization. Another, more rational solution, is choosing a possible reorganization method based on specific criteria. Such criteria for the modeling of microstructure evolution during forming processes are the shape and sizes of the CA space (or cells) and the number of grains. A block diagram of the automatic selection of the space reorganization method based on the set of decision rules and current microstructure evolution state is shown in Figure 17.

Subsequently, the reorganization method can be chosen automatically in accordance with the following set of decision rules:

(1) $$\begin{array}{l} \left(n_{g}>n_{g\max}\right)\wedge \left(a_{x}=\max\left(a_{x},a_{y},a_{z}\right)\right)\Rightarrow HX\\ \left(n_{g}>n_{g\max}\right)\wedge \left(a_{y}=\max\left(a_{x},a_{y},a_{z}\right)\right)\Rightarrow HY\\ \left(n_{g}>n_{g\max}\right)\wedge \left(a_{z}=\max\left(a_{x},a_{y},a_{z}\right)\right)\Rightarrow HZ\\ \left(a_{x}>3a_{z}\right)\Rightarrow BXZ\\ \left(a_{x}>2a_{z}\right)\wedge \left(n_{g}>2n_{g\min}\right)\Rightarrow HX\\ \left(a_{y}>2a_{z}\right)\wedge \left(n_{g}>2n_{g\min}\right)\Rightarrow HY \end{array}$$

where: *n_g_*—number of grains, *n_g_*_min_ and *n_g_*_max_—minimal and maximal number of grains, *a_x_*, *a_y_*, and *a_z_*—cellular automata space sizes, *BXZ*—cellular space length reduction (*a_x_*/2) and thickness bonding (2*a_z_*), *HX*—length halving (*a_x_*/2), HY—width halving (*a_y_*/2) and HZ—thickness halving (*a_z_*/2).

The condition for the application of cellular automata space straightness option is the selected level of shear strains where at least one of these strains is greater than the predetermined value γ_cr_: max (|γ_xy_|, |γ_xz_|, |γ_yz_|)> γ_cr_.

## 4. Modeling of Flat and Shape Rolling Processes

**Figure 18:**
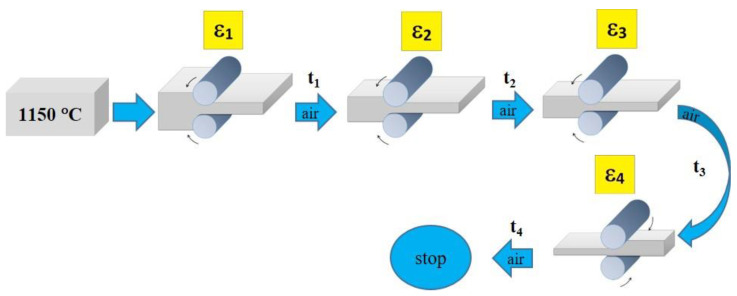
Flat rolling scheme.

**Figure 19:**
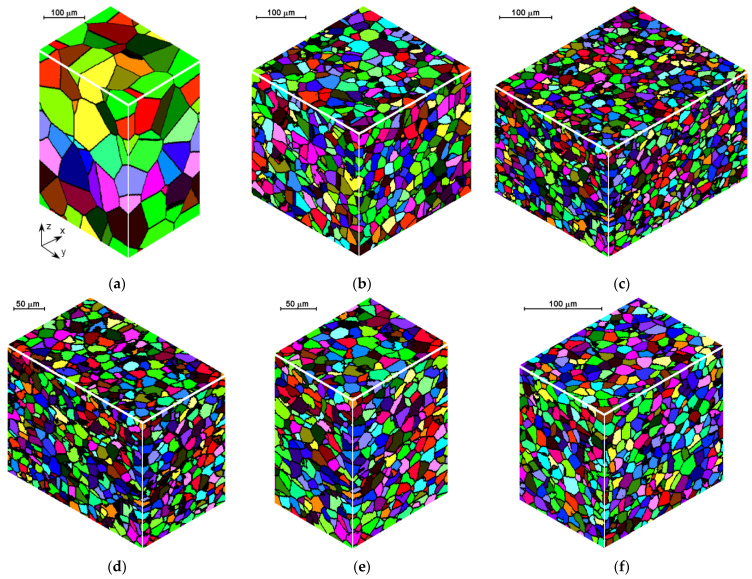

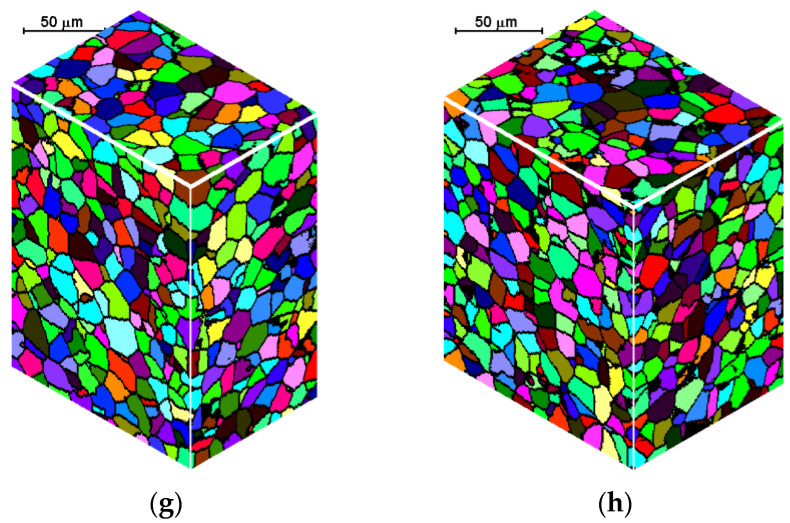
Microstructures: (**a**) initial, (**b**) before 2nd pass, (**c**) before 3rd pass, (**d**) before 3rd pass and after the modeled space reduction—Cut X, (**e**) before the 3rd pass and after the modeled space reduction—Cut Y, (**f**) before the 4th pass, (**g**) before the 4th pass and after the modeled space reduction—Cut X, (**h**) after the 4th pass and cooling.

**Figure 20:**
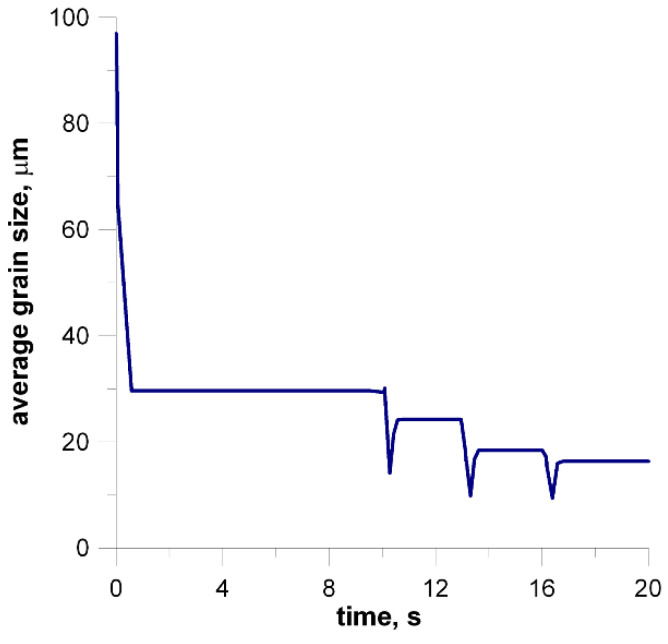
Changes in average grain size.

**Figure 21:**
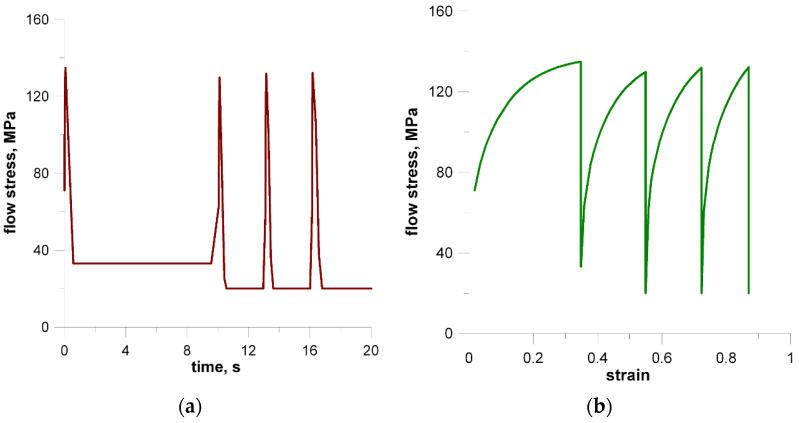
Flow stress changes in relation to time (**a**) and strain (**b**).

To demonstrate the use of the methods presented in the previous sections two processes are considered and some simulation results are presented. Flat rolling is the first process. The description of the material model can be found in the papers published in the previous years [29,30]. The description of the microstructure evolution model during the flat rolling process adapted for AISI 304L steel can be found elsewhere [31]. A database of materials developed during the subsequent work and the possibility of selecting various variants of analytical dependencies, defining, i.e., the average size of the recrystallized grain, enables the application of the model, in principle, to any material and various parameters of the process. This allows good modeling of the flat rolling process. The general modeling scheme of flat rolling considered in the context of the application of an algorithm for the automatic selection of space reorganization methods is presented in Figure 18 and the parameters used in the simulations are presented in Table 1. The specimens of air-cooled material were rolled in four passes. The temperature at the beginning of the process was 1150 °C. The FCA modeling was applied to the rolling of the sheet with a thickness of *g* = 17.5 mm and length of *l* = 120 mm, rolls diameter *d* = 250 mm, and rotational rolls speed of *n* = 50 rpm. The material microstructure evolution was simulated in the sheet axis and static recrystallization was modeled. At the start of the process, the initial microstructure with periodic boundary conditions was created as in Figure 19a. FCA space contains 200 × 200 × 200 cells with the representative volume *V_r_* = 0.2 × 0.25 × 0.35 = 0.0175 mm^3^. The initial microstructure includes 60 grains with an average grain size of about 97 μm. The *x*-axis has the rolling direction, *y* moves widthwise and *z* along the thickness axis. The size of CA in the *y*-direction remains unchanged with *x* size direction increasing and *z* direction decreasing. The simulation results are presented as isometric images with the microstructure at selected moments (Figure 19b–h) as plots of average grain size changes (Figure 20). Flow stress in relation to time and strain are presented in Figure 21a,b, respectively. The description of the dislocation density and flow stress model can be found in the published papers [29,31]. The model was developed based on the Taylor dislocation theory [32]. It is used for the determination of flow stress, which can be written as a function of average dislocation density:

(2) $$\sigma =\sigma_{0}+\alpha \mu b\sqrt{\rho_{av}}$$

where: *σ*_0_—stress necessary to move the dislocation in the absence of other dislocations, *α*—coefficient, *μ*—shear modulus, *b*—the magnitude of the Burgers vector, *ρ_av_*—average dislocation density.

**Figure 22:**
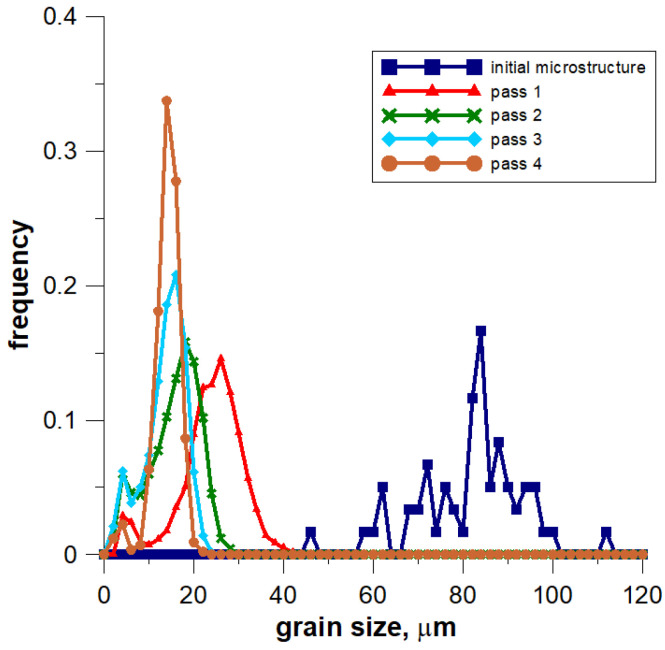
Grain size distribution for the modeled passes of the flat rolling process.

The model of the evolution of the dislocation density is applied for each grain independently and then average dislocation density is calculated considering grains dislocation densities and their volumes. Figure 22 shows the grain size distribution for the modeling passes. During the simulations, space was reorganized automatically using the development set of decision rules to select reorganization options (conditions (1)). As can be seen, cutting was used after a certain number of passes. Taking into account flow stress–time and flow stress–strain plots, it can be seen that the strain accumulated in the material is insufficient to complete recrystallization in the interpass time between pass 1 and 2. The reference value of flow stress is obtained for passes 2–4 so the recrystallization is complete.

**Figure 23:**
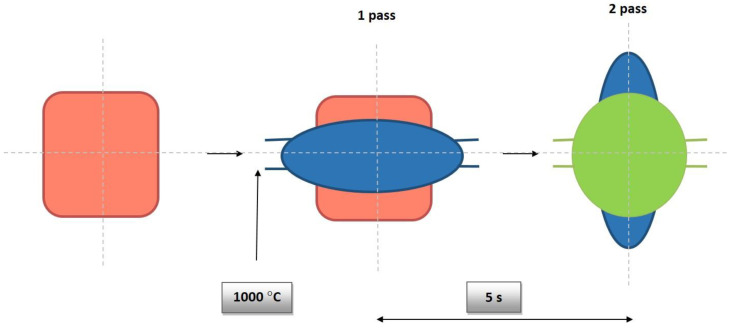
Modeling scheme of the shape rolling.

**Figure 24:**
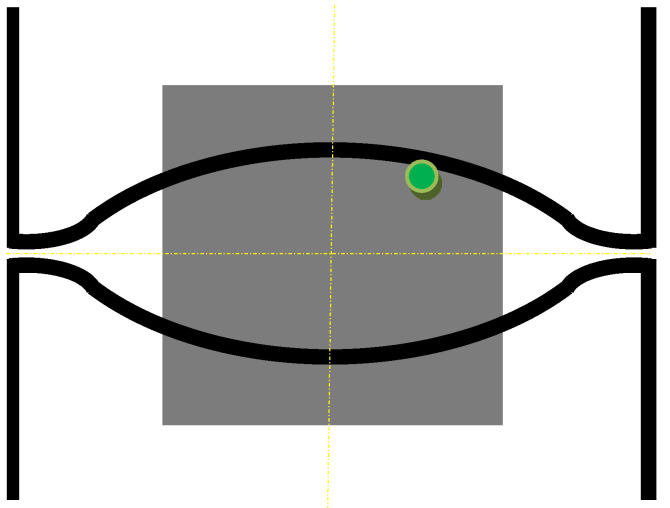
Location of modeling point.

**Figure 25:**
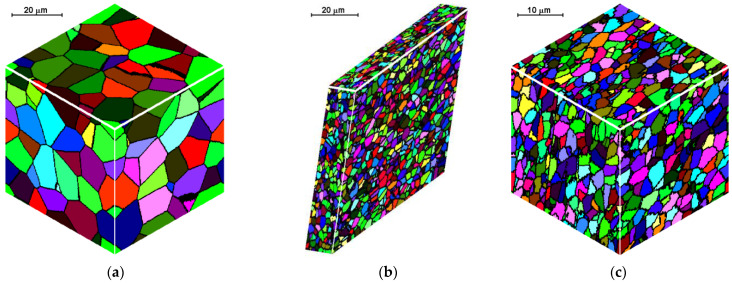

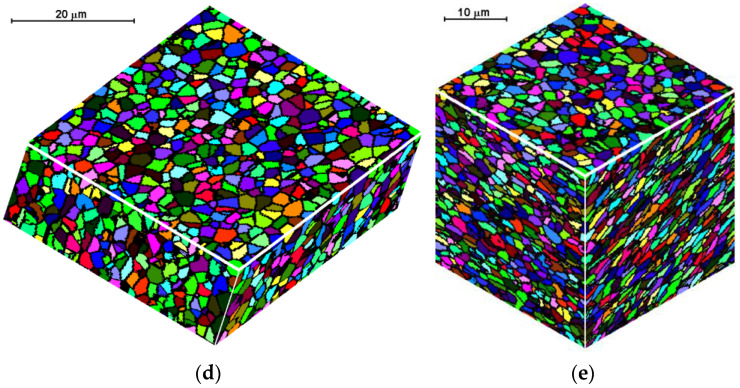
Microstructures: (**a**) initial, (**b**) before 2nd pass, (**c**) before 2nd pass and after straightening, (**d**) final microstructure, (**e**) final microstructure after straightening.

**Figure 26:**
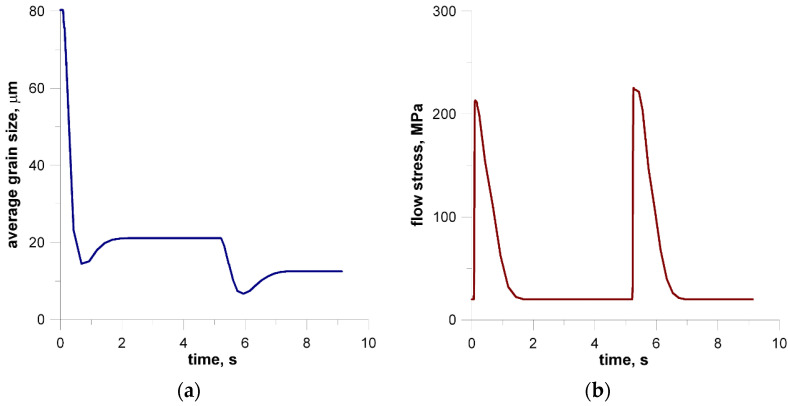
Average grain size–time (**a**) and flow stress–time (**b**) changes during shape rolling.

**Figure 27:**
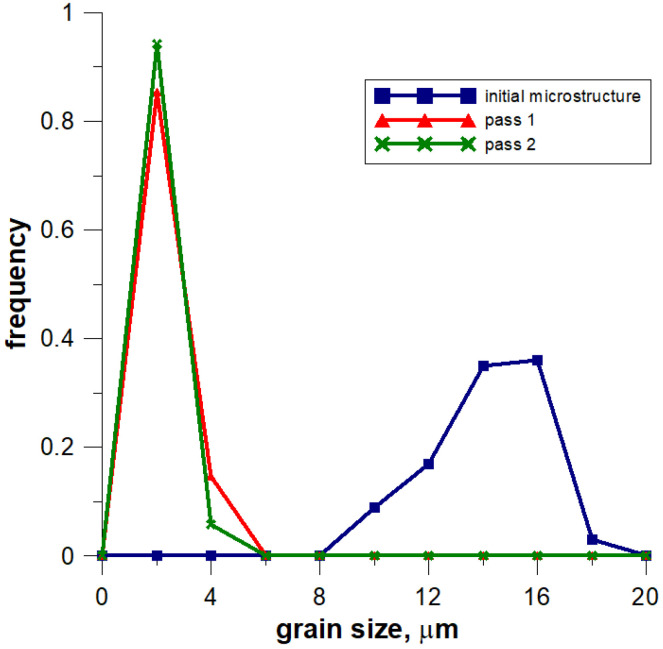
Grain size distribution for the modeled passes of the shape rolling process.

The second modeled example is the shape rolling process. The schemes of the mutual use of CA and FEM methods, according to their role in the model, can be divided into two groups. The first one contains solutions when the simulations are carried out independently, without any feedback. The systems with the cooperation of both components can be included in the second group. Then, one component is primary, another is secondary, which serves to deliver the parameters and variables for the proper calculations by the primary method. CA can be secondary, only provided that it is of a simple structure and does not require high calculation requirements, such as memory and calculation time. Cellular automata cannot be used for simulation of microstructure evolution as a secondary method owed to the computational requirements, especially for 3D tasks. For the simulation of the microstructure evolution in this process, an independent variant of modeling without feedback with the finite element method was used. In this case, postprocessing takes place to obtain information about the microstructure. The first stage of the simulation consists of the modeling of the shape rolling process with the use of the FEM method. FEM modeling allows determining the basic process parameters: temperature, components of strain and strain rate tensors, strain rate intensity at arbitrary points of deformed material. Some of these parameters (time, temperature and components of strain rate tensor) were used as input data for modeling microstructure evolution by frontal cellular automata. Simulation of microstructure evolution with this approach can be performed at any point (for which data can be obtained) using any Finite Element Method (FEM) code. Two passes of rolling according to the square–oval–round scheme were modeled and static recrystallization was considered. Figure 23 shows the shape rolling scheme used in the modeling. The cuboid sample with dimensions of 15 × 15 × 100 mm^3^ was used in the FEM simulation of the rolling in the rolls with a diameter of 200 mm and rolling speed of 0.6 m/s. The obtained sizes of the oval were 25.6 × 8.6 mm^2^, while the final round had the diameter of 12.8 mm. Several points for the simulation of microstructure evolution were selected. The results of the FEM simulation for these points were transferred to FCA. Figure 24 represents the location of one of the simulation points whose results are presented below. The calculations were carried out for the FCA space with initial dimensions of 50 × 50 × 50 μm^3^ and the space containing 8 million cells (200 × 200 × 200). The initial microstructure was created with periodic boundary conditions; the number of grains equals 100 with an average grain size of about 80 μm. The microstructure for several moments is presented in Figure 25. Changing deformation conditions have an impact on the shape of the cells and the whole cellular space. The developed algorithm for the “straightening” of FCA space with significant cell distortions was used (as per (1)). The plots of changes of average grain size (Figure 26a) and flow stress with time (Figure 26b) are presented. The grain size distribution for all the analyzed passes is shown in Figure 27. It can be seen that the reference value of flow stress is obtained for all passes (Figure 26b), so the recrystallization is complete. Taking into account the grain size distributions the results are more focused on some interval, which defines directly the average value of grain size.

## 5. Summary

The paper presents two very important issues related to the modeling of plastic processes by cellular automata, i.e., the CA geometry and boundary conditions. There is a direct relationship between them because the boundary condition is a result of the selected geometry of the space. Different types of boundary conditions applicable to the unchanged or varying topology of cellular automata are presented, i.e., open (including semi-open), periodic (closed), periodic with displacement, and combined. In general, simulations of the microstructure evolution in different processes are mainly realized with the use of the periodic conditions and many of them are discussed in the literature. The presented semi-open, periodic with displacement and combined conditions are new propositions of solutions, which can be applied to the modeling of multistage deformation processes. Taking into account the obtained simulation results, conclusions can be drawn regarding the use of different boundary conditions. Regarding the unchanged topology, only semi-open conditions are not always suitable for the conditions imposed by the simulation because the boundary has an impact on the kinetics of microstructural phenomena. With respect to the varying topology, the periodic conditions with displacement in one direction equal to half of the space length are most useful. Combined conditions include both open (semi-open) and closed boundary conditions. Large deformation in one or multiple-stage deformation processes often leads to the distortion of the space and cells and requires space reorganization using appropriate boundary conditions. Four methods of space reorganization were developed and presented, i.e., cutting, cutting and bonding, doubling, and straightening. The developed set of decision rules for automatic selection of the reorganization method based on the process parameters and variables is presented. Process conditions have a direct impact on the use of appropriate reorganization methods. Application of reorganization methods to the modeling of flat and shape rolling processes and examples of simulation results are presented. The developed reorganization methods can be an important element used in the modeling of processes, where microstructure formation is determined by several processes occurring simultaneously (i.e., deformation, nucleation, grain growth) and the deformation is introduced to the CA space such as, for example, dynamic recrystallization. They can also be used in the analysis of sequential processes (e.g., multi-stage deformation and static recrystallization). The introduction of real deformation into the cellular automata space requires appropriate space reorganization.

## Figures and Tables

**Table 1:** Simulation parameters.

Pass Number	Temperature *T*, °C	Input Thickness *h*_0_, mm	Output Thickness *h*_1_, mm	Percentage Reduction *r*, %	Time between the Passes *τ*, s
1	1150	17.5	12.95	26	10
2	1120	12.95	10.88	16	3
3	1100	10.88	9.36	14	3
4	1080	9.36	8.24	12	4

## Data Availability

Not applicable.
